# Highly Enhanced Visible-Light-Driven Photoelectrochemical Performance of ZnO-Modified In_2_S_3_ Nanosheet Arrays by Atomic Layer Deposition

**DOI:** 10.1007/s40820-018-0199-z

**Published:** 2018-04-11

**Authors:** Ming Li, Xinglong Tu, Yunhui Wang, Yanjie Su, Jing Hu, Baofang Cai, Jing Lu, Zhi Yang, Yafei Zhang

**Affiliations:** 1 Key Laboratory for Thin Film and Microfabrication of the Ministry of Education, Department of Micro/Nano Electronics, School of Electronics, Information and Electrical Engineering, Shanghai Jiao Tong University, Shanghai, 200240 People’s Republic of China; 2 State Key Lab of Transducer Technology, Shanghai Institute of Microsystem and Information Technology, Chinese Academy of Sciences, Shanghai, 200050 People’s Republic of China; 3 National Engineering Research Center for Nanotechnology, Shanghai, 200241 People’s Republic of China; 4 College of Science, Nanjing University of Posts and Telecommunications, Nanjing, 210023 People’s Republic of China

**Keywords:** In_2_S_3_/ZnO, Heterojunction, Nanosheet arrays, Atomic layer deposition, Photoelectrochemical, Water splitting, Energy band

## Abstract

Photoanodes based on In_2_S_3_/ZnO heterojunction nanosheet arrays (NSAs) have been fabricated by atomic layer deposition of ZnO over In_2_S_3_ NSAs, which were in situ grown on fluorine-doped tin oxide glasses via a facile solvothermal process. The as-prepared photoanodes show dramatically enhanced performance for photoelectrochemical (PEC) water splitting, compared to single semiconductor counterparts. The optical and PEC properties of In_2_S_3_/ZnO NSAs have been optimized by modulating the thickness of the ZnO overlayer. After pairing with ZnO, the NSAs exhibit a broadened absorption range and an increased light absorptance over a wide wavelength region of 250–850 nm. The optimized sample of In_2_S_3_/ZnO-50 NSAs shows a photocurrent density of 1.642 mA cm^−2^ (1.5 V vs. RHE) and an incident photon-to-current efficiency of 27.64% at 380 nm (1.23 V vs. RHE), which are 70 and 116 times higher than those of the pristine In_2_S_3_ NSAs, respectively. A detailed energy band edge analysis reveals the type-II band alignment of the In_2_S_3_/ZnO heterojunction, which enables efficient separation and collection of photogenerated carriers, especially with the assistance of positive bias potential, and then results in the significantly increased PEC activity.

**Electronic supplementary material:**

The online version of this article (10.1007/s40820-018-0199-z) contains supplementary material, which is available to authorized users.

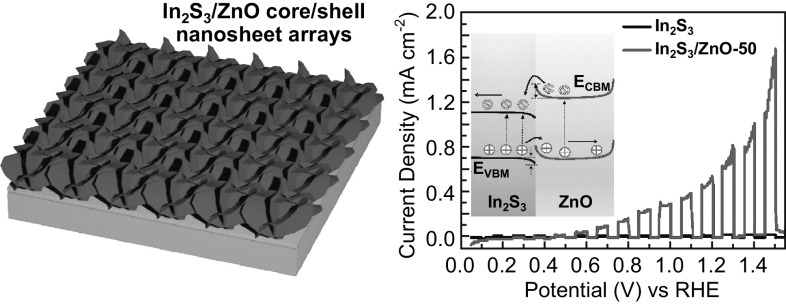

## Highlights

- The In_2_S_3_/ZnO core/shell nanosheet arrays (NSAs) were fabricated by atomic layer deposition of ZnO over In_2_S_3_ NSAs, demonstrating highly enhanced photoelectrochemical performance for water splitting.
- The In_2_S_3_/ZnO NSAs exhibit an optimal photocurrent of 1.64 mA cm^−2^ and incident photon-to-current efficiency of 27.64%, which are 70 and 116 times higher than those of the pristine In_2_S_3_ NSAs, respectively.
- A detailed energy band edge analysis reveals the type-II band alignment of the In_2_S_3_/ZnO heterojunction.

## Introduction

Photoelectrochemical (PEC) water splitting is regarded as one of the most attractive approaches for producing hydrogen in a clean, renewable, and eco-friendly manner to store solar energy, which has aroused significant interest in the recent years [1–5]. To efficiently convert the abundant solar energy into a storable and high-energy–density chemical energy, H_2_, it is desirable to pursue and design a suitable semiconductor photoelectrode satisfying the stringent requirements of wide-range absorption, high carrier mobility, long carrier lifetime, and high stability [6, 7]. However, there is no single one material that can satisfy all the aforementioned requirements among more than about 130 types of semiconductor materials [6]. To address these challenges, nanostructured architectures have been explored because of their various advantages compared to bulk materials [8–11]. Alongside the recent population of graphene, two-dimensional (2D) nanostructures, such as nanosheets, nanoplates, and nanoflakes, especially vertical nanoarray structures, are of special interest in artificial photosynthesis owing to their unique mechanical, physical, and chemical properties, as well as extremely large surface areas [12–14].

Among the known nanostructured semiconductors, metal chalcogenides have attracted substantial attention as a group of highly efficient photocatalysts for PEC water splitting [15]. As one of the most important III–VI chalcogenides, indium sulfide (In_2_S_3_) has been well studied for its applications in photocatalysts, solar cells, and other optoelectronic devices [16–20]. The defect spinel structure β-In_2_S_3_, which is an *n*-type semiconductor with a bandgap of 2.0–2.3 eV, has been reported to be a promising photoanode material for PEC water splitting under visible-light irradiation in all three different crystal structures owing to its relatively negative conduction band edge, moderate charge transport properties, stable chemical, and physical characteristics along with low toxicity [20–22]. To date, β-In_2_S_3_ nanocrystals with various 2D morphologies, such as nanosheets, nanoplates, nanoflakes, and nanobelts, have been successfully synthesized by different methods as photoanode materials for PEC applications [23–26]. However, the PEC performance of pure In_2_S_3_ nanocrystals themselves remains far from satisfactory. As an efficient strategy for improving the PEC conversion efficiency, elemental doping (Co and Zr) has been adopted to modify the electronic structure of 2D In_2_S_3_ nanocrystals as photocatalysts [23, 26]. Whereas the fabrication of photoelectrodes typically includes a process of coating the synthesized nanocrystals onto conductive substrates such as fluorine-doped tin oxide (FTO) glasses, it results in deceased effective area for photon capturing and a hindered direct pathway for charge transfer and collection because the nanostructures can hardly refrain from agglomeration and re-stacking [6, 14]. In addition, it is challenging to establish good ohmic contact between the conductive substrate and the deposited nanosheet-based film by the solution processed fabrication approach, which impedes the rapid transport of electrons and then increases the charge recombination. All of the above will undoubtedly limit further improvement in PEC performance for 2D In_2_S_3_ nanocrystal-based photoanodes.

It has been demonstrated that constructing nanoarray structures such as nanosheet arrays (NSAs) is an efficient way to avoid the abovementioned limitations and then further enhance the PEC properties of semiconductor photoelectrodes [27–30]. The architectures can exploit all of the advantages of 2D nanocrystals due to their intrinsic merits of elevated light absorptance, shortening minority carrier diffusion and increased electrode/electrolyte interface compared to a film photoelectrode [6, 14]. Furthermore, the heterojunction photoelectrodes consisting of two or more dissimilar semiconductors exhibit more advantages over those made from single semiconductors in PEC water splitting [31]. The heterojunction photoelectrodes can not only improve photogenerated carrier separation and transfer for directional face-to-face migration, but also enhance optical absorption and chemical stability by choosing a corrosion resistive material to interface with electrolytes [32–34]. For the In_2_S_3_ NSAs, the construction of 2D heterojunctions with other semiconductors would be an effective way to further elevate the PEC conversion efficiency. Although a ZnO layer has been coated onto In_2_S_3_ NSAs by magnetron sputtering to improve the PEC activity in our recent work, the further PEC performance enhancement is still hindered by the formed nonconformal In_2_S_3_/ZnO interfaces [34–36].

Herein, we report a remarkable enhancement of PEC performance for the In_2_S_3_ NSAs by constructing a heterojunction with ZnO. In particular, the ZnO overlayer was uniformly coated onto the solvothermal-grown In_2_S_3_ NSAs by an atomic layer deposition (ALD) method. The enhanced optical and PEC performance of In_2_S_3_/ZnO heterojunction NSAs has been optimized by controlling the thickness of the ZnO overlayer. Furthermore, we analyze the energy band structure of In_2_S_3_/ZnO heterojunction to illustrate the mechanism behind the dramatically improved PEC activity.

## Experimental Procedure

### Growth of In_2_S_3_ NSAs on FTO Glasses

A facile solvothermal process was introduced to the growth of In_2_S_3_ NSAs on FTO glasses. Typically, a cleaned FTO substrate, angled against the vessel wall and facing down, was put into a Teflon autoclave containing 40 mL InCl_3_·4H_2_O (24 mM) and thioacetamide (63 mM) ethylene glycol solution. After reacting at 200 °C for 2 h, a canary yellow film grew on the surface of FTO as shown in Fig. S1, indicating the formation of In_2_S_3_ NSAs.

### Deposition of ZnO onto In_2_S_3_ NSAs

**Fig. 1:**
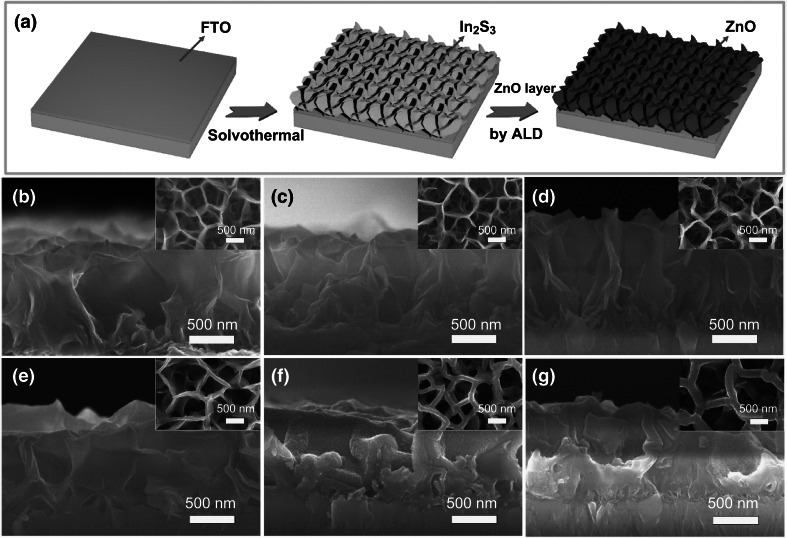
**a** Schematic illustration of the preparation of In_2_S_3_/ZnO NSAs. Cross-sectional SEM images of the In_2_S_3_/ZnO-*x* NSAs with different ZnO overlayer thicknesses: **b–g** 0, 5, 10, 20, 50, and 100 nm, respectively. Insets: the corresponding top-view SEM images

The ZnO overlayer was deposited on the In_2_S_3_ NSAs by the ALD method as shown in Fig. 1a. One ALD cycle of ZnO deposition included four processes: 0.1-s pulse of diethylzinc, 3-s purge with N_2_, 0.1-s pulse of H_2_O, and 4-s purge with N_2_. The thickness of ZnO (0.2 nm/cycle) was controlled by the cycle number. The deposition temperature was 150 °C. The products were labeled as In_2_S_3_/ZnO-*x* NSAs, where *x* represents the thickness (nm) of the ZnO shell layer.

### Characterization

A field emission scanning electron microscope (FE-SEM, Ultra 55, Carl Zeiss, Germany) operating at 20 kV was used to observe the morphology and surface topography of the nanostructured films. The microstructures were characterized by a transmission electron microscope (TEM, Talos F200X, FEI, USA) operating at 200 kV. The crystalline structures were analyzed by X-ray diffraction (XRD, D8 ADVANCE, Bruker, Germany) with Cu K_α_ radiation (*λ* = 0.154056 nm) at a voltage of 40 kV and current of 40 mA. The transmission, reflection and absorption spectra were determined by a UV–Vis-NIR spectrophotometer (Lambda 950, PerkinElmer, USA). The ultraviolet photoelectron spectroscopy (UPS) measurements were carried out using a spectrometer (Axis Ultra DLD, Shimadzu, Japan) with a He I line (21.22 eV).

### PEC Measurements

A PEC test system was used to characterize the PEC properties; it was composed of an electrochemical station (CHI 650E, Shanghai Chenhua, China) and a solar simulator (CHF-XM500, Beijing Perfectlight, China) equipped with a 500-W Xenon lamp and an AM 1.5-G filter. The sample, Pt mesh, and Ag/AgCl (saturated KCl) electrode were treated as the working, counter, and reference electrodes, respectively, and a 1.0 M KCl aqueous solution was used as the electrolyte. The electrochemical impedance spectra (EIS) were carried out with frequencies ranging from 100 kHz to 0.1 Hz under a sinusoidal perturbation with 5 mV amplitude. The Mott–Schottky plot was performed with a frequency of 1 kHz under an AC amplitude of 10 mV. The measured potentials versus Ag/AgCl were converted to a reversible hydrogen electrode (RHE) scale via the Nernst equation (Eq. 1):

1 $$E_{\text{RHE}} = E_{\text{Ag/AgCl}} + \, 0.059{\text{pH + }}E_{0}$$

where *E*_RHE_, *E*_Ag/AgCl_, and *E*_0_ are the converted potential versus RHE, the experimental potential measured against the Ag/AgCl reference electrode, and the standard potential of Ag/AgCl (saturated KCl) at 25 °C (i.e., 0.197), respectively.

## Results and Discussion

Figure 1b shows the cross-sectional and top-view SEM images of the as-grown In_2_S_3_ nanostructural film on the FTO substrate through a facile solvothermal process. Obviously, the In_2_S_3_ film is constructed by vertically oriented and interconnected 2D nanosheets, which exhibit smooth surfaces and graphene-like morphologies. The film thickness and nanosheet size are about 1.1 μm and 603 nm, respectively. The XRD pattern (Fig. S2a) suggests that the weak peak appearing at 47.9° can be indexed to the (-440) crystal plane of cubic β-In_2_S_3_ (JCPDS No. 32-0456) [23, 25] and reveals the low crystallinity of the nanostructural In_2_S_3_ film. The energy-dispersive X-ray spectroscopy spectrum of the In_2_S_3_ NSAs (Fig. S2b) shows that the atomic ratio of S and In elements is about 1.66, which is close to the stoichiometric ratio of In_2_S_3_ (S/In = 1.5). To fabricate heterojunction NSAs, the In_2_S_3_ nanosheets were conformably coated with ZnO overlayers through a thermal ALD process at 150 °C (Fig. 1a). Figure 1c–g shows the cross-sectional and top-view SEM images of the In_2_S_3_/ZnO core/shell NSAs with varied shell thicknesses. It can be observed that the shell thickness increases with increasing deposition cycle and the morphology of NSAs remains essentially. This confirmed a uniform and conformal ZnO deposition process.

**Fig. 2:**
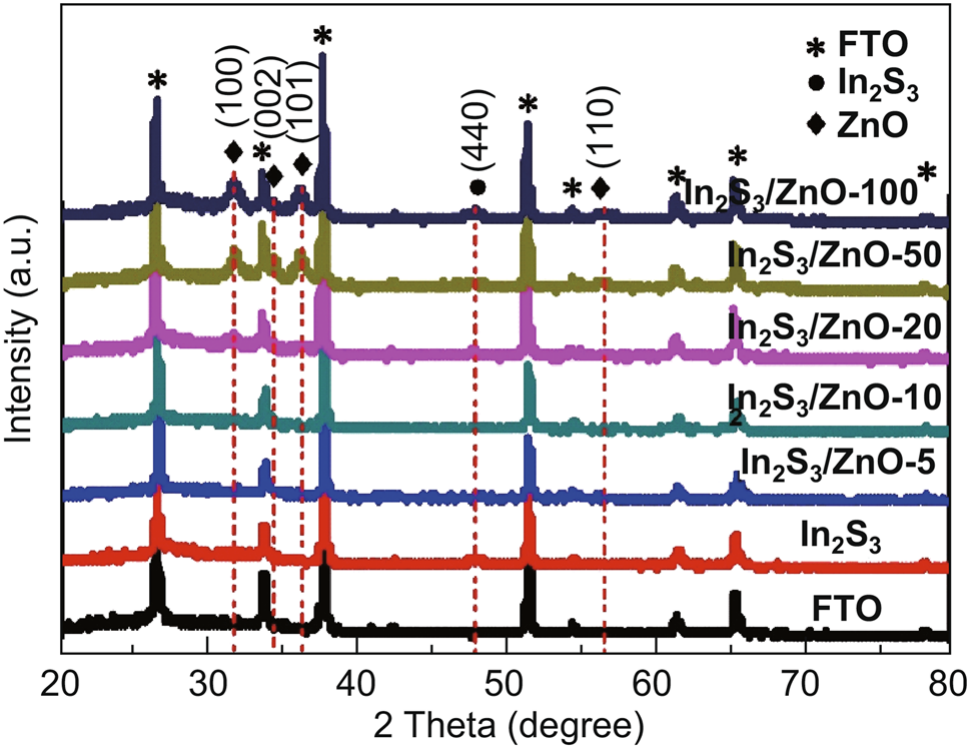
XRD patterns of the In_2_S_3_/ZnO-*x* NSAs compared to those of the FTO substrate and pristine In_2_S_3_ NSAs

As shown in Fig. 2, the XRD patterns of the In_2_S_3_/ZnO-x NSAs were characterized and compared to those of the FTO substrate and pristine In_2_S_3_ NSAs. After subtracting the background from FTO and In_2_S_3_, the characteristic diffraction peaks centered at 31.7°, 34.4°, 36.3°, and 56.6° can be well indexed to the (100), (002), (101), and (110) planes of hexagonal ZnO (JCPDS No. 36-1451), respectively. The intensity of ZnO diffraction peaks increases with increasing thickness of the ZnO overlayer. Noticeably, the In_2_S_3_/ZnO-5 and In_2_S_3_/ZnO-10 samples did not show distinct XRD peaks of ZnO owing to the ultrathin shell thickness.

**Fig. 3:**
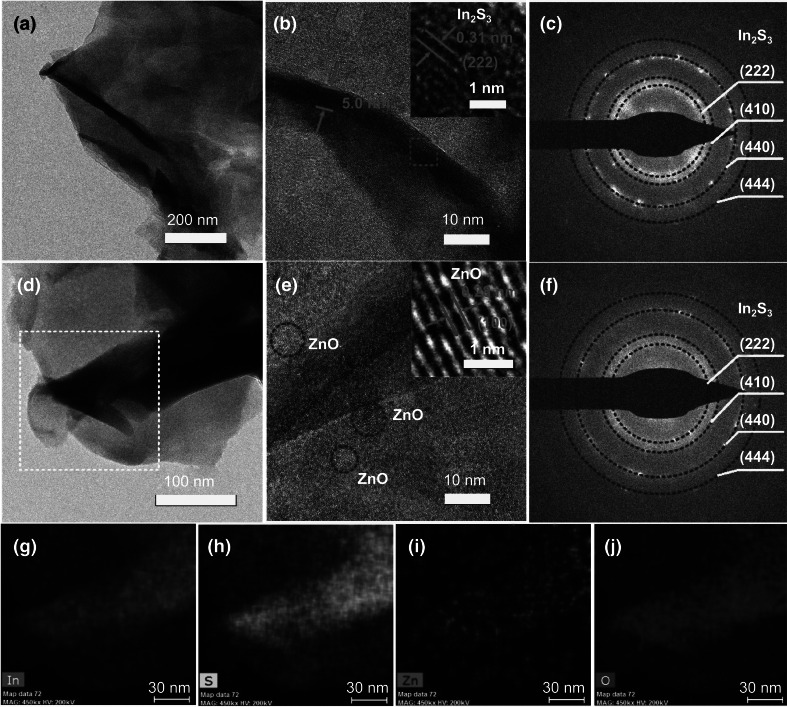
TEM characterization of the In_2_S_3_ and In_2_S_3_/ZnO-5 nanosheets: **a** Low-magnification and **b** high-magnification TEM images, **c** SAED pattern of the In_2_S_3_ nanosheets, inset: HRTEM image, **d** low-magnification and **e** high-magnification TEM images, **f** SAED pattern of the In_2_S_3_/ZnO-5 nanosheets, inset: HRTEM image, and **g–j** element mapping of In, S, Zn, and O, respectively, for the area of the white dotted box of In_2_S_3_/ZnO-5 nanosheets shown in **d**

TEM characterization was used to present the microtopography and microstructure of the In_2_S_3_/ZnO core/shell nanosheets, which further confirms the modification of the ZnO overlayer on In_2_S_3_. As shown in Fig. 3a, the In_2_S_3_ nanosheets connecting with each other exhibit 2D graphene-like morphology. The In_2_S_3_ nanosheets showing a thickness as low as ~ 5 nm (Fig. 3b) are constructed by nanocrystals as confirmed by the corresponding selected area electron diffraction (SAED) pattern (Fig. 3c). The cross-sectional HRTEM image of an In_2_S_3_ nanosheet (inset in Fig. 3b) shows that the fringe spacing of 0.31 nm matches well with the interplanar spacing of (222) planes, indicating that the ultrathin In_2_S_3_ nanosheets possess preferentially exposed (222) facet [23, 25]. This is consistent with the XRD characterization result, as the {-440} planes are perpendicular to the {222} planes for cubic In_2_S_3_. After the deposition of the 5-nm ZnO layer by ALD, the sample still conserves its nanosheet morphology as shown in Fig. 3d. The HRTEM image shown in Fig. 3e confirms the coat of ZnO nanocrystals on the surfaces of In_2_S_3_ nanosheets. The typical HRTEM image (inset in Fig. 3e) demonstrates that the deposited ZnO shows a lattice spacing of ~ 0.29 nm corresponding to the interplanar distance of the (100) crystal plane of hexagonal ZnO, which also verifies the higher diffraction peak belonging to (100) plane observed in XRD pattern (Fig. 2). The electron diffraction spot of ZnO is hardly distinguished from those of the In_2_S_3_ matrix because there is a relatively small amount of ZnO (Fig. 3f). Besides, the element mapping for a part of the composite nanosheet (Fig. 3g–j) further proved the uniform distribution of ZnO on In_2_S_3_ nanosheets.

**Fig. 4:**
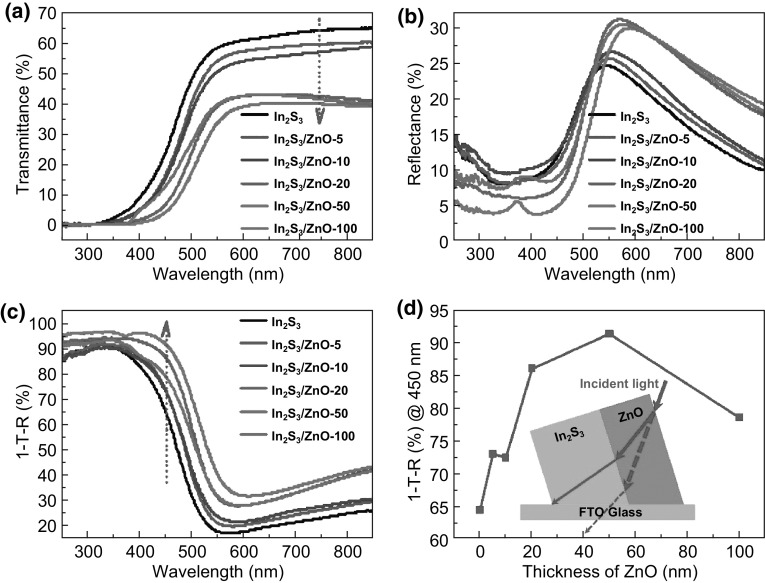
**a** Transmission, **b** reflection, **c** absorption spectra, and **d** the absorptance at 450 nm as a function of the ZnO thickness for the In_2_S_3_/ZnO-*x* NSAs

The transmittance (*T*) and reflectance (*R*) were measured to investigate the influence of the ZnO overlayer on the optical properties of the composite NSAs (Fig. 4a, b). The absorptance (*A*) was obtained according to the relationship *T* + *R* + *A* = 1. As shown in Fig. 4c, the absorptance increases with an increase in the thickness of the ZnO shell layer and reaches a maximum at a thickness of roughly 50 nm in the entire measured wavelength region of 250–850 nm. Furthermore, the In_2_S_3_/ZnO composite NSAs also exhibits a broadened absorption range and induces a red shift of the absorption edge when compared to the pristine In_2_S_3_ NSAs. As illustrated in Fig. 4d, the absorptance at 450 nm for the In_2_S_3_ NSAs has been enhanced from 64.2 to 91.1% after the modification of the 50-nm ZnO shell layer, but it decreases to 78.3% as the shell thickness further increases to 100 nm. The influence of the ZnO layer on the optical properties of In_2_S_3_ NSAs includes the following three aspects: First, the ZnO layer can prolong light transportation distance in the nanostructured film to enhance light absorption because of its relatively smaller refractive index compared to In_2_S_3_ (inset in Fig. 4d) [37–39]. Second, the grown ZnO film itself possesses good light absorption ability in the short wavelength region (Fig. S3a) owing to its relatively large bandgap (Fig. S3b). As a result, increasing the thickness of the ZnO shell layer is beneficial for enhancing the absorptance in this region. Lastly, however, a very thick ZnO layer will destroy the nanoarray morphology, which is not good for light trapping and results in decreased light absorption.

**Fig. 5:**
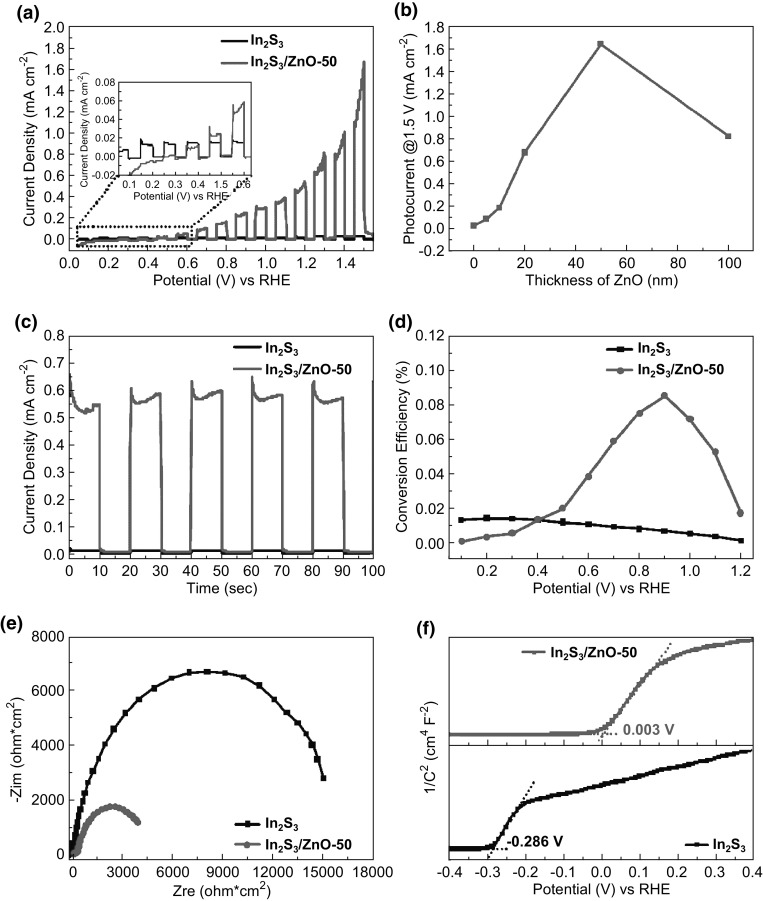
**a** LSV curves of the pristine In_2_S_3_ and In_2_S_3_/ZnO-50 NSAs under chopped AM 1.5-G simulated solar illumination, **b** photocurrent of In_2_S_3_/ZnO-x NSAs at 1.5 V versus RHE as a function of the thickness of ZnO overlayer, **c** amperometric *I*-*t* curves, **d** photoconversion efficiency versus applied bias potential curves, **e** Nyquist plots, and **f** Mott–Schottky plots of the pristine In_2_S_3_ and In_2_S_3_/ZnO-50 NSAs

Figure 5a presents a typical linear sweep voltammetry (LSV) curve of the In_2_S_3_/ZnO-50 NSAs under chopped AM 1.5-G simulated solar illumination that is compared with that of the bare In_2_S_3_ NSAs. Apparently, the nanostructured In_2_S_3_ photoanode demonstrated remarkably improved PEC activity after forming an *n*–*n*-type heterojunction with the grown ZnO layer, and the improvement increases with an increase in positive bias. Additionally, the PEC activity of the In_2_S_3_/ZnO-50 NSAs is much higher than that of the 50-nm ZnO film deposited on FTO glass by ALD (Fig. S3c). It can be seen that the composite photoanode exhibits an absolute photocurrent density of 1.642 mA cm^−2^ at 1.5 V versus RHE, which is about 70.2 and 12.2 times larger than those of the pristine In_2_S_3_ NSAs (0.0234 mA cm^−2^) and ZnO-50-nm film (0.135 mA cm^−2^) counterparts, respectively. To investigate the influence of the thickness of the ZnO shell layer on PEC performance, the LSV curves of In_2_S_3_/ZnO NSAs with varied ZnO thicknesses were characterized (Fig. S4), and the relationship between the photocurrent density at 1.5 V versus RHE and the thickness of ZnO are presented in Fig. 5b. It can be observed that the photocurrent of the nanostructured photoanode first increases with increasing thickness of the ZnO shell layer and then achieves a maximum value of 1.642 mA cm^−2^ for the In_2_S_3_/ZnO-50 NSAs. The optimal performance is comparable with those of ZnO-based nanostructured photoanodes [11, 29]. However, further increasing the thickness of ZnO overlayer to 100 nm will result in relatively suppressed photocurrent. The reasons can be partly ascribed to the deteriorated light absorption and decreased surface area for charge separation and interfacial redox reactions. Furthermore, a very thick ZnO layer will also increase the possibility for the recombination of photogenerated carriers. Figure 5c shows a comparison of the transient current density at 1.23 V versus RHE under chopped illumination for the In_2_S_3_/ZnO-50 NSAs and that of the pristine In_2_S_3_ NSAs, demonstrating its good switching behavior as a photoanode and further proving the greatly enhanced photocurrent density. The photoconversion efficiency (*η*) can be calculated with Eq. 2:

2 $$\eta \, = I\left( {1.23 \, - V_{\text{RHE}} } \right)/P_{\text{in}}$$

where *I*, *V*_RHE_ (V vs. RHE), and *P*_in_ are the photocurrent density, bias voltage, and incoming light flux (100 mW cm^−2^ for AM. 1.5-G illumination), respectively [6]. The photocurrent density at a specific bias voltage can be obtained according to Fig. 5a. Figure 5d presents the plots of photoconversion efficiency versus applied bias potential for the pristine In_2_S_3_ and In_2_S_3_/ZnO-50 NSAs. The optimal conversion efficiency of the In_2_S_3_/ZnO-50 NSAs is 0.085% at 0.9 V versus RHE, which is 6.5 times larger than that of the pristine In_2_S_3_ NSAs (0.013% at 0.2 V vs. RHE).

To explore the mechanism behind this dramatically improved PEC activity, the EIS spectrum of the In_2_S_3_/ZnO-50 NSAs was performed under AM 1.5-G illumination and compared to that of the pristine In_2_S_3_ NSAs. As shown in Fig. 5e, the semicircle diameter at high frequencies for each Nyquist plot means the charge transfer resistance (*R*_ct_), which presents the charge transfer kinetics at the electrode/electrolyte interfaces [10]. The *R*_ct_ of the In_2_S_3_/ZnO-50 NSAs under illumination is much smaller than that of the bare In_2_S_3_ NSAs photoanode, suggesting that the deposited ZnO shell layer on In_2_S_3_ nanosheets can promote charge transfer from the nanostructured photoanode to the electrolyte. As a result of the formation of the heterojunction, the photocurrent density was significantly increased.

Figure 5f presents the Mott–Schottky plots of the pristine In_2_S_3_ and In_2_S_3_/ZnO-50 NSAs, in which 1/*C*^2^ is plotted against the applied bias potential. The positive slope of the plots reveals the *n*-type semiconductor nature of the In_2_S_3_ NSAs as photoanode materials [21, 23]. The flat-band potential (*E*_FB_) can be estimated from the extrapolation of the linear region of the plots, and the *E*_FB_ of the bare In_2_S_3_ and In_2_S_3_/ZnO-50 NSAs is − 0.286 and 0.003 V versus RHE, respectively. The result confirms the positively shifted onset potential for In_2_S_3_/ZnO-50 NSAs compared to the bare In_2_S_3_ NSAs as illustrated in the inset of Fig. 5a. The reason may be correlated with the fact that the relatively thick ZnO shell itself shows a more positive onset potential than the pristine In_2_S_3_ NSAs (Figs. S3c and S4a).

**Fig. 6:**
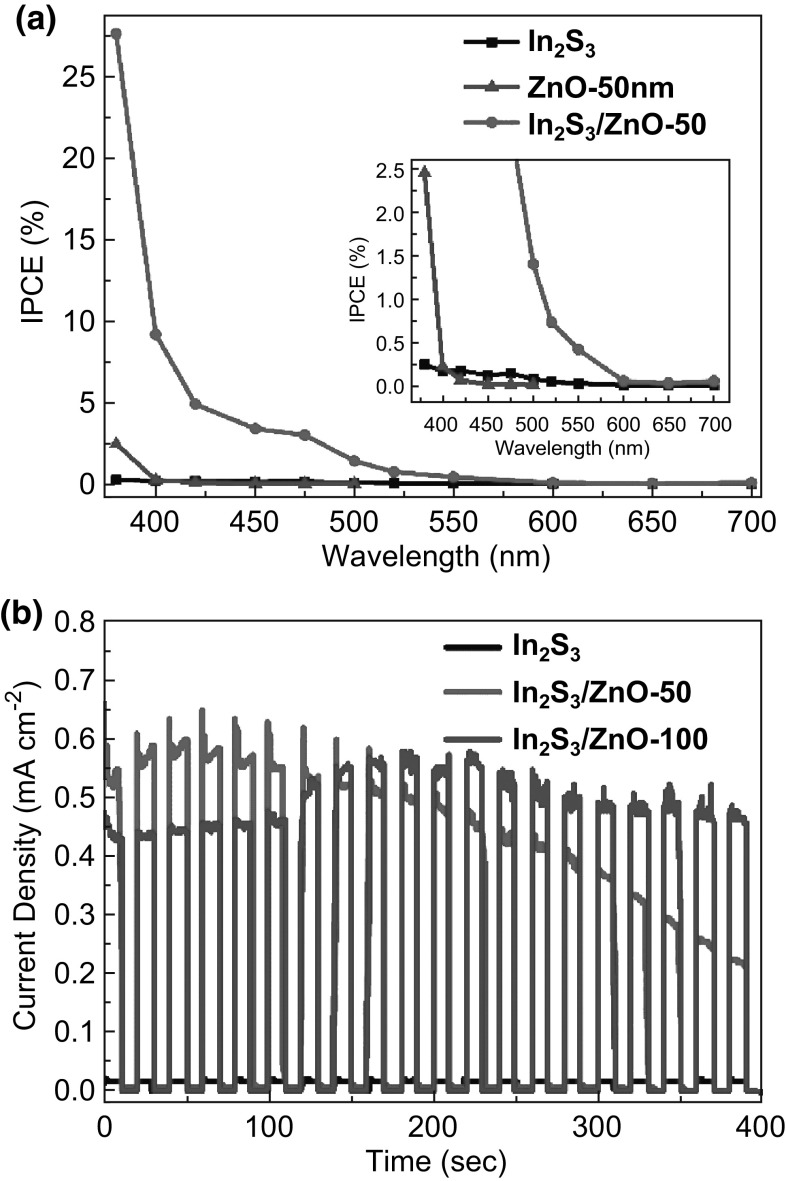
**a** IPCE of the pristine In_2_S_3_ NSA, ZnO-50-nm film, and In_2_S_3_/ZnO-50 NSAs at 1.23 V versus RHE; **b** amperometric *I*–*t* curves of the pristine In_2_S_3_, In_2_S_3_/ZnO-50, and In_2_S_3_/ZnO-100 NSAs at 1.23 V versus RHE under chopped AM 1.5-G illumination

The incident photon-to-current efficiency (IPCE) has been characterized at 1.23 V versus RHE under monochromatic irradiation from the Xenon lamp equipped with bandpass filters. It is expressed as Eq. 3:

3 $${\text{IPCE}} = \left( {1240I} \right)/\left( {\lambda P_{\text{light}} } \right)$$

where *I*, *λ,* and *P*_light_ are the photocurrent density (mA cm^−2^), the incident light wavelength (nm), and the power density of monochromatic light at a specific wavelength (mW cm^−2^), respectively [8, 9]. Figure 6a shows the IPCE spectra of the pristine In_2_S_3_ NSAs, ZnO-50-nm film, and In_2_S_3_/ZnO-50 NSAs. It can be observed that, after the modification of the ZnO overlayer, the nanostructured photoanode shows remarkably enhanced IPCE in the entire tested wavelength region. Furthermore, the increment in the short wavelength region is more significant than that in the long wavelength region, which can be ascribed to the relatively large bandgap for both In_2_S_3_ (2.45 eV, see Fig. S5) and ZnO (3.21 eV, see Fig. S3b). More specifically, the In_2_S_3_/ZnO-50 NSAs photoanode shows a maximum IPCE of 27.64% at 380 nm, which is 116 and 11 times higher than those of the pristine In_2_S_3_ NSAs (0.237%) and ZnO-50nm film (2.447%), respectively. As the light absorption enhancement is limited (Fig. 4c), the dramatically increased photocurrent should be mainly attributed to the formed In_2_S_3_/ZnO heterojunction, which promotes the highly efficient separation of photogenerated carriers.

As shown in Fig. 6b, the short-time photocurrent stability of the photoanodes was evaluated by chronoamperometric measurements at 1.23 V versus RHE under chopped illumination over 400 s. Although the In_2_S_3_/ZnO-50 NSAs exhibit much higher photocurrent density than the bare In_2_S_3_, they show relatively deteriorated photocurrent stability. The photocurrent of In_2_S_3_/ZnO-50 NSAs decreases from an initial value of 0.549 to 0.212 mA cm^−2^ after the stability test. Although the bare In_2_S_3_ NSAs demonstrate nearly unchanged photocurrent in the whole short-time test process, it can be deduced that the low PEC stability of the composite NSAs may result from the poor photocurrent stability of the deposited ZnO-50-nm film itself (Fig. S3d). Fortunately, a thick ZnO shell layer (100 nm) can be used to improve the PEC activity as well as maintain the relatively high photocurrent stability of the In_2_S_3_ NSAs (Fig. 6b).

As summarized in Table S1, we further listed the reported 2D nanostructured In_2_S_3_-based photoanodes for water splitting and compared them with our ZnO-functionized In_2_S_3_ NSAs by ALD [23–26, 34]. The results show that In_2_S_3_/ZnO-50 NSAs display the highest photocurrent density, which is significantly much higher than that of the pure In_2_S_3_. For one thing, the in situ grown In_2_S_3_ NSAs show good electrical contact with the conductive substrates, which reduces the possibility for the recombination of photogenerated carriers and is beneficial for the efficient electron collection. In addition, the NSAs architectures as photoelectrodes for PEC water splitting have intrinsic advantages of enhanced light absorptance, decoupling light absorption and charge collection, shortening minority carrier diffusion, and increased electrode/electrolyte interface for charge separation and interfacial redox reactions.

**Fig. 7:**
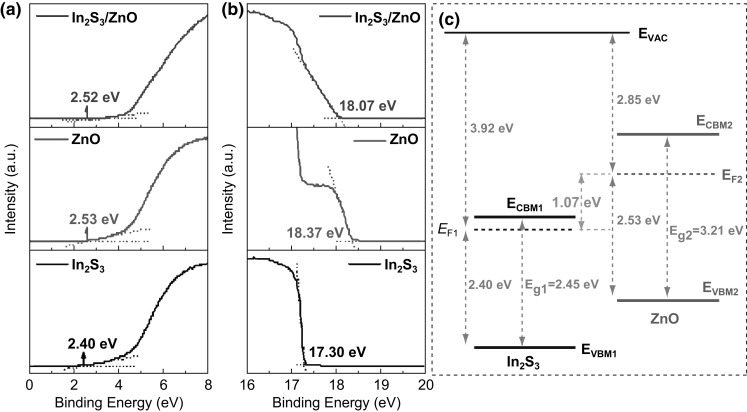
**a** Low and **b** high binding energy regions of UPS spectra for In_2_S_3_, ZnO (100 nm) and In_2_S_3_/ZnO-5. **c** Schematic band alignment for In_2_S_3_ and ZnO before the formation of heterojunction

To better understand the detailed band structure of the heterostructured nanosheets, we recorded the UPS of In_2_S_3_, ZnO, and In_2_S_3_/ZnO. Figure 7a, b presents the low and high binding energy regions of the UPS spectra, in which the low binding energy cutoff (*E*_L_) and high binding energy cutoff (*E*_H_) can be determined from the corresponding tangent line [40, 41]. As the collected electron information only comes from the sample surface with thickness about 10 atomic layers in the UPS characterization, the test results of In_2_S_3_/ZnO-5 actually correspond to those of the ZnO overlayer grown on In_2_S_3_ nanosheets. The bandgap of the pristine In_2_S_3_ nanosheets and ZnO film (2.45 and 3.21 eV, respectively) can be determined by their corresponding UV–Vis absorbance data (Figs. S3 and S5). The Fermi level (*E*_F_), valence band maximum (*E*_VBM_), and conduction band minimum (*E*_CBM_) for the samples can be calculated from the UPS data using Eqs. 4–6:

4 $$E_{\text{F}} = h\upsilon - E_{\text{H}}$$

5 $$E_{\text{VBM}} = h\upsilon - E_{\text{H}} + E_{\text{L}}$$

6 $$E_{\text{CBM}} = h\upsilon - E_{\text{H}} + E_{\text{L}} - E_{\text{g}}$$

where *hυ* (21.22 eV) is the incident photon energy. The obtained results are summarized in Table S2.

Based on the above calculated data, a schematic band alignment for In_2_S_3_ and ZnO before the formation of heterojunction can be drawn as illustrated in Fig. 7c, where *E*_VAC_ stands for the vacuum energy level. As the Fermi level of ZnO (*E*_F2_) is 1.07 eV higher than that of In_2_S_3_ (*E*_F1_), the electrons will transfer from the former to the later until the interfacial Fermi-level equalization alignment when they are subject to form a heterojunction [29]. The UPS results prove that the Fermi level of ZnO reduces from − 2.85 to − 3.15 eV after the formation of the heterojunction with In_2_S_3_.

**Fig. 8:**
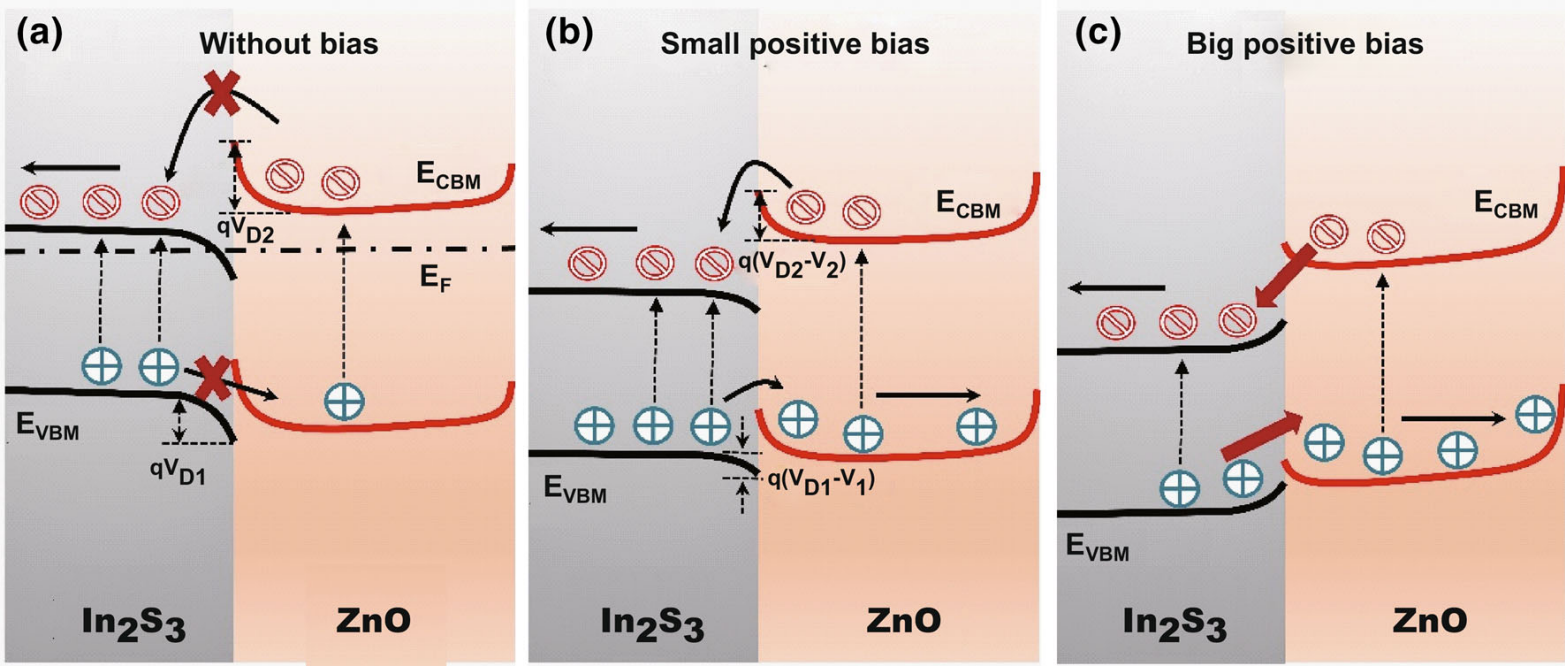
Schematic energy-level diagrams illustrating the photoactivated charge transfer processes in In_2_S_3_/ZnO heterojunction photoanode: **a** without bias, **b** with small positive bias, and **c** with big positive bias

Specifically, *E*_F1_ moves upwards along with the energy band of In_2_S_3_ at the interface, while *E*_F2_ moves downwards with that of ZnO, which results in the formation of the In_2_S_3_/ZnO heterojunction at the condition of thermal equilibrium as shown in Fig. 8a. With regard to the heterojunction interface, an accumulation layer forms on the side of In_2_S_3_ and a depletion layer on the side of ZnO, which gives rise to a built-in electric field with the direction pointing from the later to the former. The built-in potential or contact potential *V*_D_ can be expressed as Eq. 7:

7 $$qV_{\text{D}} = \, qV_{{{\text{D}}1}} + \, qV_{{{\text{D}}2}} = E_{{{\text{F}}2}} - E_{{{\text{F}}1}}$$

where *V*_D1_ and *V*_D2_ are the built-in potentials on the side of In_2_S_3_ and ZnO of the heterojunction, respectively, and q is the electron charge. The built-in potential brings about accessional potential energy for electrons at every position in the space charge region. Specifically, the energy bands of In_2_S_3_ bend downwards, and the bending amount for *E*_VBM_ and *E*_CBM_ at the heterojunction interface is q*V*_D1_. Similarly, the energy bands of ZnO bend upwards, and the corresponding bending amount for *E*_VBM_ and *E*_CBM_ is q*V*_D2_.

As illustrated in Fig. 8a, the photogenerated holes on the *E*_VBM_ of In_2_S_3_ need to overcome the potential barrier of q*V*_D1_ and then reach that of ZnO. Analogously, only the photogenerated electrons with a potential energy q*V*_D1_ higher than the *E*_CBM_ of ZnO can jump over the potential barrier to that of In_2_S_3_. When a positive bias potential *V* is applied on the In_2_S_3_/ZnO heterojunction (*V*_1_ and *V*_2_ for In_2_S_3_ and ZnO sides, respectively, *V* = *V*_1_ + *V*_2_) as shown in Fig. 8b, the potential barriers on the *E*_VBM_ of In_2_S_3_ and the *E*_CBM_ of ZnO will be reduced to q(*V*_D1 _− *V*_1_) and q(*V*_D2 _− *V*_2_), respectively. Therefore, the increase in positive bias potential is beneficial for the separation of photogenerated carriers at the heterojunction interfaces and then results in the enhanced photocurrent of the nanostructured photoanodes.

The above analysis is consistent with the results of PEC characterization. As demonstrated in the inset of Fig. 5a, when the positive bias is relatively low, the In_2_S_3_/ZnO heterojunction is not efficient for improving the photocurrent of the photoanode. The reason may be that there is a high potential barrier at the heterojunction interface owing to the existence of the big built-in potential *V*_D_, and the photogenerated carriers cannot be easily transported to the other side of heterojunction and then be collected for PEC water splitting. However, when a relatively larger positive bias is applied on the composite NSAs, the barrier height will be lowered greatly and the In_2_S_3_/ZnO heterojunction will promote the efficient separation of photogenerated carriers. The analysis is consistent with the phenomena that no photocurrent plateau can be seen for the composite photoanodes (Fig. 5a), which is attributed to the elevated driving force for charge transfer through ZnO with respect to enhancing anodic potential that further facilitates band bending (Fig. 8c) [42]. Additionally, the energy band of ZnO at the electrolyte interface bends upwards, leading to the formation of a built-in potential with the direction being consistent with that of the positive bias potential. This built-in potential will also promote charge separation, which becomes more pronounced upon increasing the bias potential.

## Conclusions

In conclusion, we fabricated the photoanodes based on In_2_S_3_/ZnO NSAs by ALD of a ZnO layer over In_2_S_3_ NSAs in situ grown on FTO glasses via a facile solvothermal process. It is found that the composite NSAs exhibit a broadened absorption range and increased light absorptance over a wide wavelength region of 250–850 nm compared to the pristine In_2_S_3_ NSAs. Furthermore, the In_2_S_3_/ZnO-50 NSAs show an optimal photocurrent of 1.642 mA cm^−2^ (1.5 V vs. RHE) and an IPCE of 27.64% at 380 nm (1.23 V vs. RHE), which are 70 and 116 times higher than those of the In_2_S_3_ NSAs counterpart, respectively. The significantly increased PEC performance primarily results from the important function of the In_2_S_3_/ZnO heterojunction for promoted photocarrier separation and collection. This strategy of surface functionalization using ALD-deposited layers may provide a facile route to design and fabricate high-performance photoanodes based on 2D nanoarray architectures.

## Electronic supplementary material

Below is the link to the electronic supplementary material.

Supplementary material 1 (pdf 576 kb)
