# Supplementary material for: Highly Enhanced Visible-Light-Driven Photoelectrochemical Performance of ZnO-Modified In2S3 Nanosheet Arrays by Atomic Layer Deposition
Source: Nanomicro Lett. 2018 Apr 11;10(3):45. doi: 10.1007/s40820-018-0199-z (PMC6199096; doi:10.1007/s40820-018-0199-z)
Supplement: Supplementary file 1 — Supplementary material 1 (pdf 576 kb) [file 40820_2018_199_MOESM1_ESM.pdf]

Supporting Information for

Highly Enhanced Visible-Light-Driven Photoelectrochemical Performance of ZnO Modified In_2S_3 Nanosheet Arrays by Atomic Layer Deposition

Ming Li[1,2], Xinglong Tu[1,3], Yunhui Wang[4], Yanjie Su[1,*], Jing Hu[1], Baofang Cai[1], Jing Lu[3,*], Zhi Yang[1], Yafei Zhang[1,*]

[1]Key Laboratory for Thin Film and Microfabrication of the Ministry of Education, Department of Micro/Nano Electronics, School of Electronics, Information and Electrical Engineering, Shanghai Jiao Tong University, Shanghai 200240, People's Republic of China

[2]State Key Lab of Transducer Technology, Shanghai Institute of Microsystem and Information Technology, Chinese Academy of Sciences, Shanghai 200050, People's Republic of China

[3]National Engineering Research Center for Nanotechnology, Shanghai 200241, People's Republic of China

[4]College of Science, Nanjing University of Posts and Telecommunications, Nanjing 210023, People's Republic of China

*Corresponding authors. E-mail: yanjiesu@sjtu.edu.cn (Yanjie Su), jinglu2004@163.com (Jing Lu), yfzhang@sjtu.edu.cn (Yafei Zhang)

Supplementary Figures and Tables

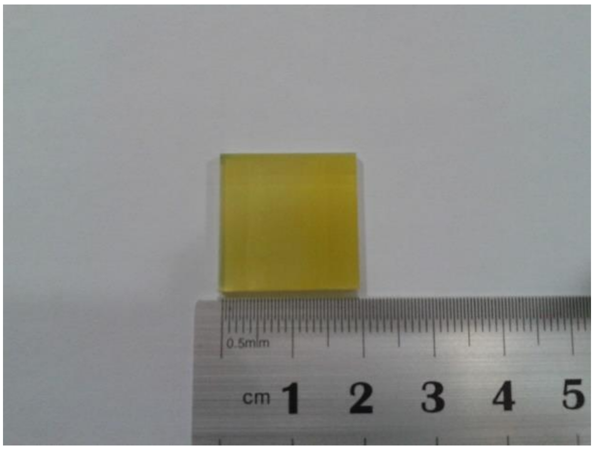

Fig. S1 Optical image of In_2S_3 NSAs grown on FTO glass substrate

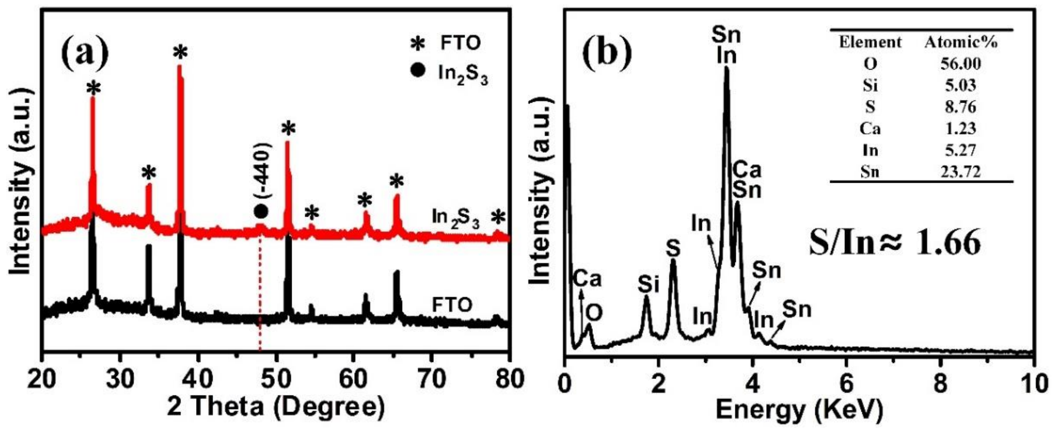

Fig. S2 a XRD pattern of In_2S_3 NSAs compared to that of the FTO substrate. **b** The typical EDS spectrum of In_2S_3 NSAs

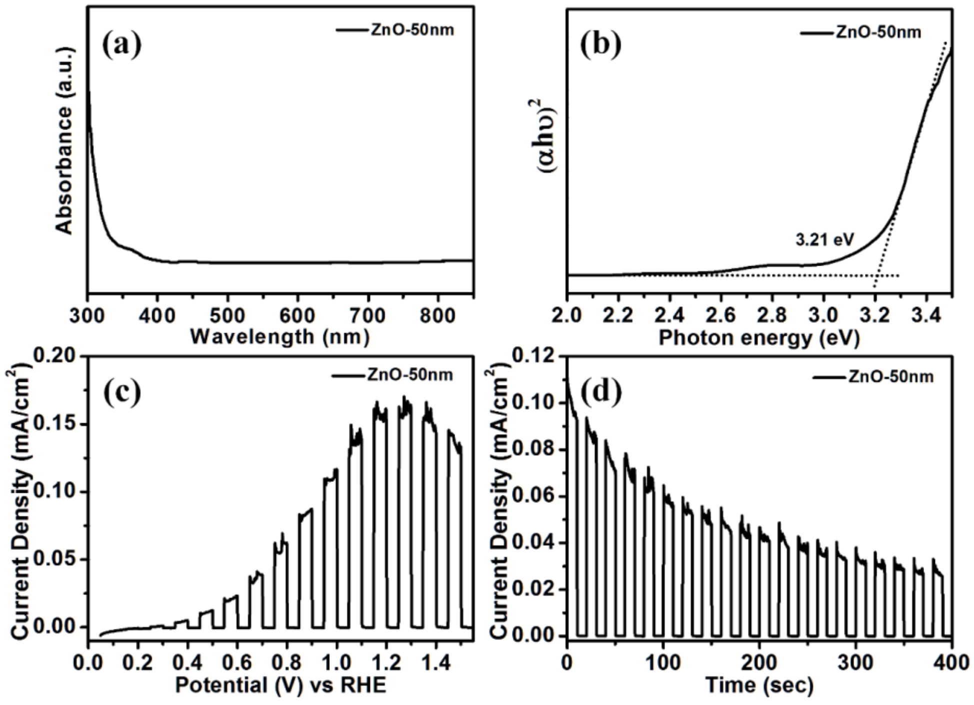

Fig. S3 a Absorbance, **b** energy band gap determination, **c** LSV curve and **d** amperometric *I-t* curve at 1.23 V_{RHE} under chopped AM 1.5G illumination for the ZnO thin film with thickness of 50 nm

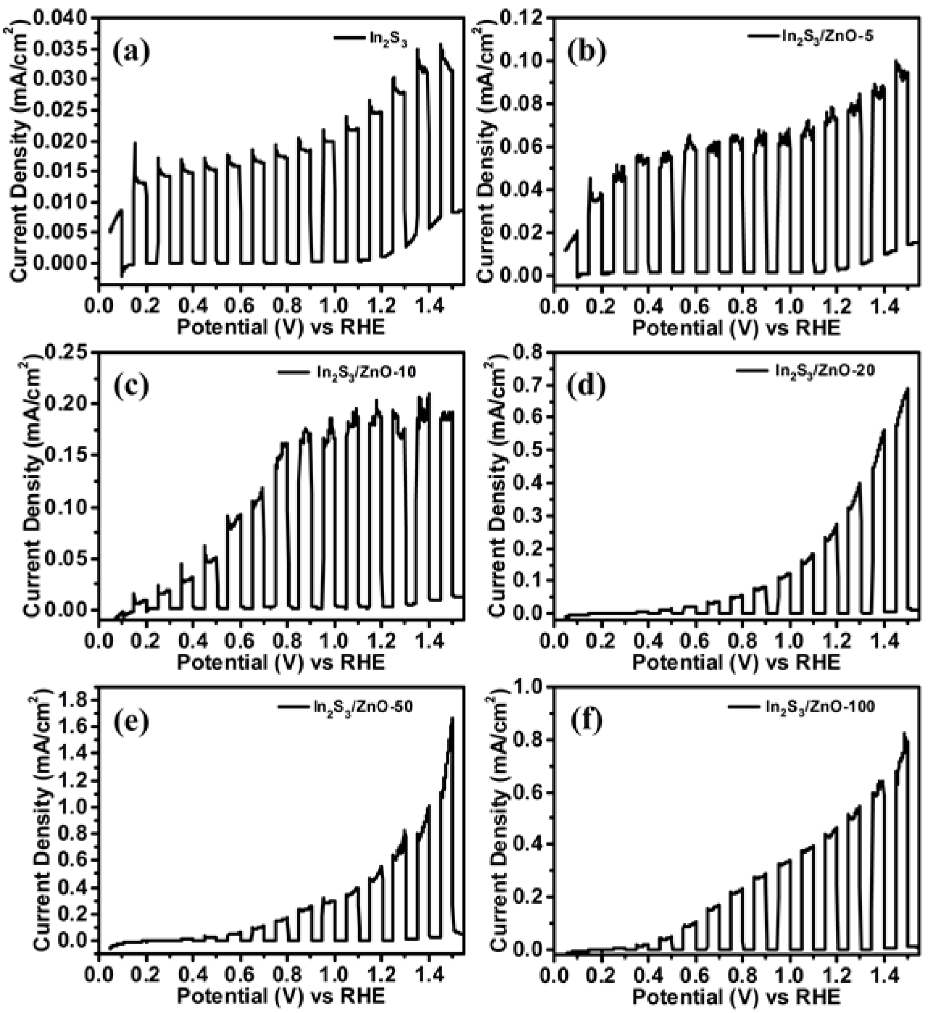

Fig. S4 LSV curves under chopped AM 1.5G illumination of the In_2S_3/ZnO-x NSAs

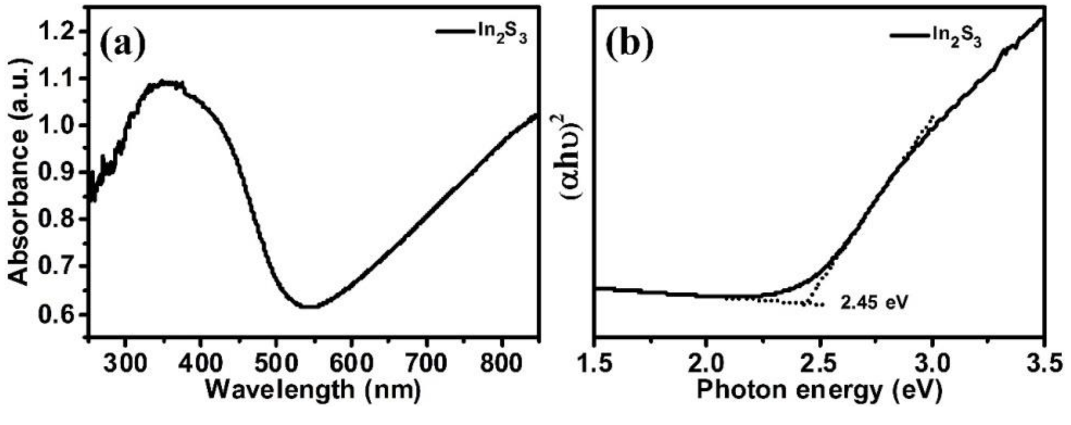

Fig. S5 a Absorbance and **b** energy band gap determination of In_2S_3 NSAs

Table S1 PEC performance of 2D nanostructured In_2S_3-based photoanodes

Photoanodes	Morphology	Photocurrent	IPCE	Reference
In_2S_3/ZnO-50	NSAs	1.64 mA cm^{-2} (1.5 V_{RHE})	27.64% @380nm (1.23 V_{RHE})	This work
In_2S_3/ZnO	NSAs	0.35 mA cm^{-2} (1.2 V_{RHE})	10.26% @380nm (1.23 V_{RHE})	[34]
Zr-doped In_2S_3	nanoflakes	1.1 mA cm^{-2} (1.3 V_{RHE})	2.5% @400nm (1.2 V_{RHE})	[23]
MoS_2-In_2S_3	nanoplates	~1 μA cm^{-2} (0.5 V_{RHE})	/	[24]
In_2S_3	nanoflakes	37 μA cm^{-2} (1.3 V_{RHE})	/	[25]
In_2S_3	nanobelts	10 μA cm^{-2} (1.3 V_{RHE})	/	[25]
Co-doped In_2S_3	nanosheets	1.17 mA cm^{-2} (1.5 V_{RHE})	46% @450nm (1.5 V_{RHE})	[26]

Table S2 Energy levels of the In_2S_3 and ZnO layers determined using UPS and UV-Vis absorption spectra, the data come from the ZnO layer for In_2S_3/ZnO-5

Sample	E_L (eV)	E_H (eV)	E_F (eV)	E_{VBM} (eV)	E_{CBM} (eV)	E_g (eV)
In_2S_3	2.40	17.30	3.92	6.32	3.87	2.45
ZnO	2.53	18.37	2.85	5.38	2.17	3.21
In_2S_3/ZnO	2.52	18.07	3.15	5.67	2.46	/
